# Multiplex Real-Time PCR-Based Newborn Screening for Severe Primary Immunodeficiency and Spinal Muscular Atrophy in Osaka, Japan: Our Results after 3 Years

**DOI:** 10.3390/genes15030314

**Published:** 2024-02-28

**Authors:** Tomokazu Kimizu, Masatoshi Nozaki, Yousuke Okada, Akihisa Sawada, Misaki Morisaki, Hiroshi Fujita, Akemi Irie, Keiko Matsuda, Yuiko Hasegawa, Eriko Nishi, Nobuhiko Okamoto, Masanobu Kawai, Kohsuke Imai, Yasuhiro Suzuki, Kazuko Wada, Nobuaki Mitsuda, Shinobu Ida

**Affiliations:** 1 Department of Pediatric Neurology, Osaka Women’s and Children’s Hospital, Izumi 594-1101, Japan; yasuzuki@wch.opho.jp; 2 Department of Neonatal Medicine, Osaka Women’s and Children’s Hospital, Izumi 594-1101, Japan; kwada@ped.med.osaka-u.ac.jp; 3 Department of Perinatal and Pediatric Infectious Diseases, Osaka Women’s and Children’s Hospital, Izumi 594-1101, Japan; 4 Department of Hematology/Oncology, Osaka Women’s and Children’s Hospital, Izumi 594-1101, Japan; yokada@wch.opho.jp (Y.O.); asawada@wch.opho.jp (A.S.); 5 Department of Laboratory Medicine, Osaka Women’s and Children’s Hospital, Izumi 594-1101, Japan; misaki98@wch.opho.jp (M.M.); hfujita@wch.opho.jp (H.F.); airie@wch.opho.jp (A.I.); idas@wch.opho.jp (S.I.); 6 Department of Medical Genetics, Osaka Women’s and Children’s Hospital, Izumi 594-1101, Japan; keiko.matuda@gmail.com (K.M.); yuhase@wch.opho.jp (Y.H.); nishieriko@gmail.com (E.N.); genetics@wch.opho.jp (N.O.); 7 Department of Pediatric Gastroenterology, Nutrition, and Endocrinology, Osaka Women’s and Children’s Hospital, Izumi 594-1101, Japan; kawaim@wch.opho.jp; 8 Department of Pediatrics, National Defense Medical College, Saitama 359-0042, Japan; koh-imai@ndmc.ac.jp; 9 Department of Maternal Fetal Medicine, Osaka Women’s and Children’s Hospital, Izumi 594-1101, Japan; nmitsuda@wch.opho.jp

**Keywords:** newborn screening, multiplex qPCR, primary immunodeficiency, spinal muscular atrophy

## Abstract

In newborn screening (NBS), it is important to consider the availability of multiplex assays or other tests that can be integrated into existing systems when attempting to implement NBS for new target diseases. Recent developments in innovative testing technology have made it possible to simultaneously screen for severe primary immunodeficiency (PID) and spinal muscular atrophy (SMA) using quantitative real-time polymerase chain reaction (qPCR) assays. We describe our experience of optional NBS for severe PID and SMA in Osaka, Japan. A multiplex TaqMan qPCR assay was used for the optional NBS program. The assay was able to quantify the levels of T-cell receptor excision circles and kappa-deleting recombination excision circles, which is useful for severe combined immunodeficiency and B-cell deficiency screening, and can simultaneously detect the homozygous deletion of SMN1 exon 7, which is useful for NBS for SMA. In total, 105,419 newborns were eligible for the optional NBS program between 1 August 2020 and 31 August 2023. A case each of X-linked agammaglobulinemia and SMA were diagnosed through the optional NBS and treated at early stages (before symptoms appeared). Our results show how multiplex PCR-based NBS can benefit large-scale NBS implementation projects for new target diseases.

## 1. Introduction

Recently, large-scale newborn screening (NBS) for new target diseases, such as primary immunodeficiency (PID), spinal muscular atrophy (SMA), and lysosomal storage disease, has been implemented in several countries due to the development of innovative testing and treatment technologies. When attempting to implement NBS for new target diseases, it is important to consider the availability of multiplex assays or other tests that can be integrated into existing systems [1]. Among the potential new target diseases of NBS, PID and SMA can be screened for simultaneously by subjecting dried blood spot (DBS) samples to quantitative real-time polymerase chain reaction (qPCR) assays.

PID is characterized by an increased susceptibility to infections, autoimmunity, autoinflammatory diseases, allergies, bone marrow failure, and malignancy. PID is currently categorized into 10 groups and 485 diseases, based on a genetic classification system [2]. Severe combined immunodeficiency (SCID) is caused by a spectrum of genetic defects and is the most severe type of PID. Its most common feature is the absence of T-cells or T-cells only being present in very low numbers, accompanied by the absence of B lymphocytes and natural killer cells or such cells being non-functional. SCID patients are born asymptomatic but experience repeated severe or opportunistic infections within the first six months of life. They are sometimes vulnerable to secondary infections caused by live vaccines. They generally die before the age of 1 year unless they receive adequate curative treatment. This includes hematopoietic stem cell transplantation (HSCT) and, in selected genetic forms of SCID, enzyme replacement therapy or gene therapy [3].

B-cell deficiency (BCD) is characterized by a lack of B lymphocytes and results in a severe antibody deficiency. X-linked agammaglobulinemia (XLA) is major type of BCD, which is commonly caused by a mutation in or deletion of the *BTK* gene that prevents the normal development of B lymphocytes. BCD patients are asymptomatic after birth but experience repeated infections. The early diagnosis of XLA and the administration of immunoglobulin infusion therapy result in better outcomes [4].

T-cell receptor excision circles (TRECs) are small circular DNA fragments formed through the rearrangement of the T-cell receptor α locus and do not multiply during cell division [5]. TREC quantification can be used as a neonatal screening tool for detecting SCID [6]. In the process of B-cell maturation, immunoglobulin kappa-deleting recombination excision circles (KRECs) are produced during kappa-deleting recombination allelic exclusion and isotypic exclusion of the λ chain [7]. KREC quantification is useful for identifying early B-cell maturation defects and can be used to screen for BCD [8]. Since the first report of TREC-based NBS in the United States in 2005 [9], the practicality and clinical utility of TREC-based screening have been demonstrated in many studies. In 2018, all 50 states in the United States adopted TREC as a tool for NBS for T-cell deficiency, and since then most cases of SCID in the United States are detected via NBS [10]. It has also been reported that NBS using KREC is being carried out in various regions around the world [11,12,13,14]. We implemented screening for severe forms of PID in Osaka using KREC- and TREC-based methods, making us one of the first groups in Japan to use this NBS technique.

SMA is an autosomal recessive neuromuscular disorder, which is characterized by progressive muscle atrophy. SMA is caused by mutations in the survival motor neuron 1 (*SMN1*) gene, encoding the survival motor neuron (SMN) protein, which was shown to map to chromosome 5q13 in 1995 [15]. About 95% of patients with SMA exhibit homozygous deletion of *SMN1*. *SMN2*, which differs from *SMN1* by only a few nucleotides in exon 7, also maps to chromosome 5q13. The number of copies of *SMN2* is considered to be a major modifying factor of the SMA phenotype, as most SMA patients with higher *SMN2* copy numbers show milder SMA phenotypes [16]. *SMN1* and *SMN2* copy number analysis based on the multiplex ligation-dependent probe amplification (MLPA) test is the standard method used to diagnose SMA and determine the optimal treatment strategies for patients with SMA. The incidence rate of SMA is 1 in 10,000 live births, and about half of these cases involve SMA type 1, which is a severe early infantile onset form in which the patient usually carries two copies of *SMN2* [17]. Patients with SMA type 1 never achieve independent sitting, and the majority die before the age of 2 years without medical support [18]. New innovative drugs for SMA have recently been developed, and early treatment with these drugs has been shown to be important for improving the prognosis of SMA patients [19,20]. In studies in which presymptomatic SMA patients that were presumed to have severe disease and, hence, were not expected to ever achieve independent ambulation without early treatment, were treated with these drugs, it was reported that none of the patients required invasive respiratory management, and approximately 80% of them achieved independent ambulation [21,22,23]. In Japan, nusinersen (Spinraza^®^, an antisense oligonucleotide), onasemnogene abeparvovec (Zolgensma^®^, a gene replacement therapy), and risdiplam (Evrysdi^®^, a small molecule) have been approved as drugs for SMA. As of October 2023, nusinersen and onasemnogene abeparvovec have been approved for the presymptomatic treatment of genetically diagnosed SMA patients in Japan. Thus, newborn screening for SMA (SMA-NBS) is now strongly recommended, and its implementation in each prefecture is rapidly expanding in Japan.

In a study of DBS samples, Taylor JL et al. reported a multiplex qPCR TREC assay that can simultaneously determine the presence or absence of *SMN1* in 2015 [24]. As this assay allows SMA and SCID to be screened for simultaneously and at low cost, the implementation of SMA-NBS has rapidly expanded in the US since 2018, when SMA was added as a core condition by the Recommended Uniform Screening Panel (RUSP). Moreover, a successful pilot trial of SMA-NBS using a similar multiplex qPCR assay was reported in Taiwan [25]. These trials showed that SMA-NBS may be added to SCID-NBS at low cost by using a multiplex qPCR assay, and performing simultaneous testing for SCID reduces the cost of SMA-NBS by approximately half compared with testing for SMA alone. In addition, in a previous study screening for PID was reported to be possible by simultaneously measuring the levels of TREC and KREC in DBS samples using a multiplex qPCR assay [8].

The universal NBS program in Japan started in 1977. The use of tandem mass analyzers for the universal NBS program was introduced nationwide from 2014. As of 2024, the NBS program, which requires mandatory written consent, covers 20 diseases as a public health program. An optional fee-based NBS program for new target diseases has been gradually introduced in several prefectures in Japan since around 2015. In Osaka, we started an optional NBS program based on a multiplex qPCR assay of TREC, KREC, and SMN1 as a pioneering initiative in Japan in August 2020. By November 2023, optional NBS for PID and SMA had already been implemented in 38 of the 47 prefectures in Japan. Currently, a multiplex qPCR assay of TREC, KREC, and *SMN1* is widely employed in these prefectures. In this study, we show the merits of using multiplex qPCR assays of TREC, KREC, and SMN1 for large-scale NBS implementation projects, based on our experiences during the three years of the optional NBS program for PID and SMA in Osaka, Japan. The stepwise addition of new target diseases for multiplex qPCR assay-based NBS, which is described below, is considered to be a strategy that would be useful in many countries and regions.

## 2. Materials and Methods

### 2.1. Optional Screening for PID and SMA in Osaka

Osaka Women’s and Children’s Hospital (OWCH), which consists of a perinatal center, children’s hospital, and a universal NBS center, was founded in 1981 in Osaka, Japan. The center routinely screens 70% of the newborns in Osaka Prefecture (60,000 live births/year).

In August 2020, an optional NBS screening program for PID was started. The program, which required a fee (JPY 5500; about USD 40) to be paid, was based on, but separate from, the existing universal NBS system run at OWCH. Then, an optional NBS program for SMA with no additional fee was started in October 2021 after a 9-month pilot study. Due to the critical role played by prenatal providers in the uptake of optional fee-based screening, we carefully and repeatedly educated them about target diseases for NBS, such as the importance of early diagnosis and treatment for patients with these diseases, at the start of the optional NBS program.

### 2.2. Consent for the Optional NBS Program of OWCH

Obstetricians in Osaka provide information about the optional NBS program for PID and SMA to all parents by giving leaflets to their families and uploading the information onto the internet. The leaflet contains the URL of the homepage of the optional NBS program, which provides additional information.

If an infant’s parents request participation in the optional program, their obstetrician needs to obtain informed consent for this from them before any DBS samples obtained from their newborn are sent to OWCH.

### 2.3. Workflow for the Optional NBS Program

#### 2.3.1. From Collecting DBS Samples to Performing the Screening Tests

DBS samples were collected at maternity hospitals within 4–6 days after birth, sent to OWCH on day 5–9, and tested on day 6–13.

In the screening tests, DNA was automatically extracted from a 3 mm punch from a DBS sample using Magtration technology [26]. Then, a multiplex TaqMan qPCR assay was used for the optional NBS tests. The assay for TREC and KREC used for PID screening can also simultaneously detect homozygous deletion of *SMN1* exon 7, which is used to screen for SMA. The details of each assay have been described previously [6,8,25].

**Figure 1:**
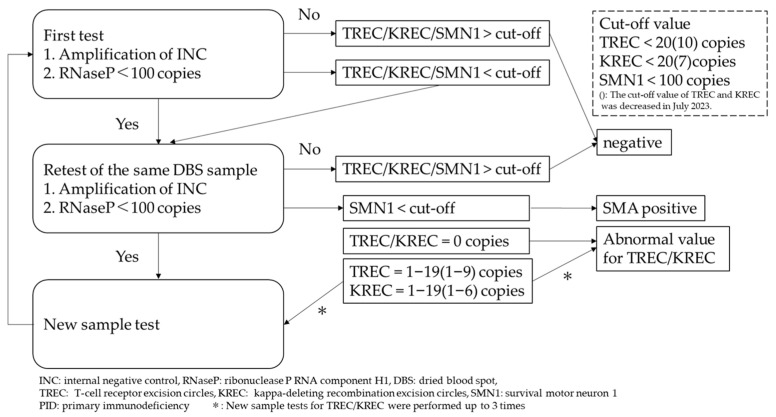
Validation process for our NBS testing.

Ribonuclease P RNA component H1 (RNase P) was used as an internal reference control for this assay. DBS samples from which <100 copies/mL of RNase P can be amplified are considered to be invalid. The validation process for our NBS testing is described in Figure 1.

#### 2.3.2. Workflow for the PID-NBS Program

The cut-off values for TREC/KREC were adjusted during the study period based on the number of samples with abnormal values and their outcomes in order to avoid unnecessary recalls. By changing the cut-off value, the proportion of retests that required a new sample remained at around 0.3~0.5%. During the first period, a cut-off value of 20 copies/μL of TREC or KREC was applied. As relatively high numbers of term infants with abnormal TREC and KREC values were seen, the cut-off value for TREC was decreased to 10 copies/μL, and that for KREC was reduced to 7 copies/μL, in July 2023. Abnormal TREC and/or KREC values (suspected lymphopenia) were defined as shown in Figure 1. If the TREC and/or KREC copy number was lower than the relevant cut-off value, a new blood sample was collected and retested. This process was repeated up to three times depending on the clinical condition. Further examinations of patients showing abnormal TREC and/or KREC values were performed at OWCH. In cases of T-cell lymphopenia, the presence/absence of the thymus was checked. Children that were diagnosed with T-cell or B-cell deficiency underwent a genetic investigation, and the parents were counseled by medical geneticists, before undergoing appropriate therapeutic interventions delivered by immunologists or hematologists.

#### 2.3.3. Workflow for the SMA-NBS Program

The workflow for the SMA-NBS program was the same as that described in a previous study [27]. In short, a positive screening result was defined as the detection of <100 copies/µL of *SMN1* (Figure 1). Positive results were obtained within about 14 days after birth, and a pediatric neurologist at OWCH notified the parents of babies with suspected SMA of the result on the same day. At the first visit, information, mainly about SMA and genetic tests for definitively diagnosing SMA, was provided to the parents, and genetic testing for SMA with an MLPA assay for definitive diagnosis was performed with a newly collected blood sample. Through this workflow, most babies that are diagnosed with SMA due to the NBS can receive specific treatments based on our therapeutic proposals within 30 days after birth.

## 3. Results

**Figure 2:**
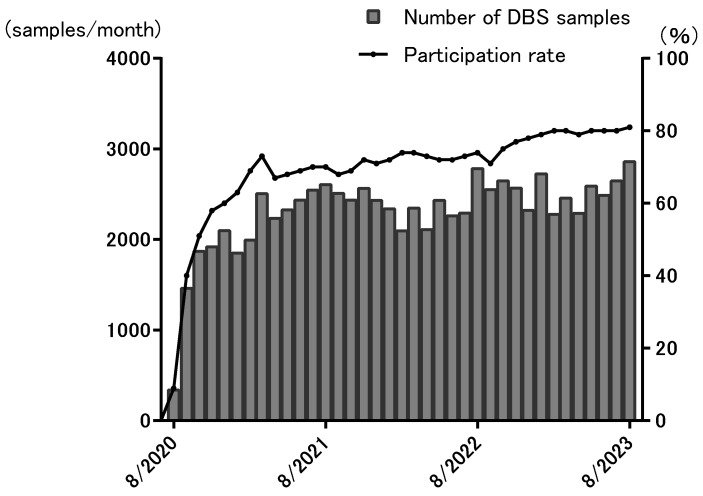
Results of the optional newborn screening conducted in Osaka.

The optional NBS program run at OWCH requires a separate interfacility agreement with the maternity hospitals in Osaka from the universal NBS program. At the beginning of August 2020, the optional NBS-contracted hospitals accounted for 29% of the universal NBS-contracted hospitals, but as of August 2023, this had increased to 86%. Currently, the optional NBS program run at OWCH covers about 80% (36,000 births/year) of the babies that undergo universal NBS (40,000 births/year) in Osaka. Therefore, about 3000 DBS samples per month are sent from maternity hospitals to OWCH (Figure 2).

SMA-NBS was additionally implemented from 1 October 2021. There was no change in the number of DBS samples sent from maternity hospitals to OWCH due to the use of a multiplex qPCR assay for the simultaneous screening for PID and SMA. The participation rate of the universal NBS program in Japan is 100% in all prefectures.

**Figure 3:**
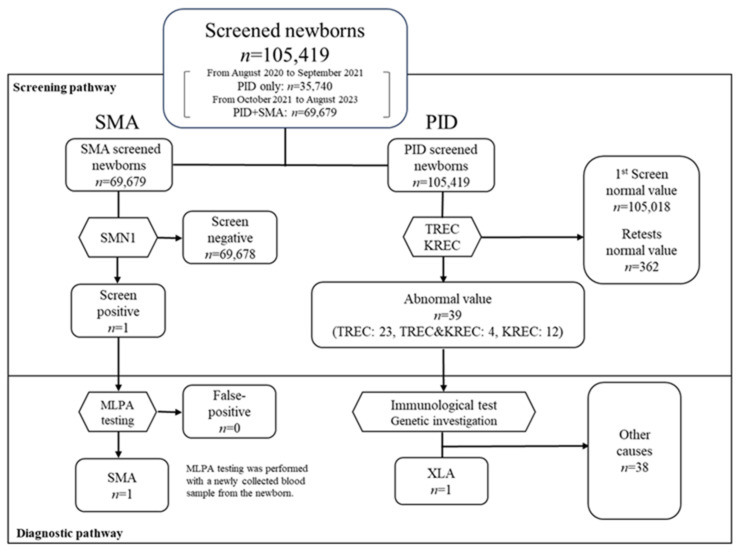
Results of the SMA and PID screening.

In total, 105,419 newborns were eligible for the optional NBS program conducted at OWCH from 1 August 2020 to 31 August 2023 (Figure 3). From 1 August 2020 to 30 September 2021, 35,740 newborns were screened for PID only. From 1 October 2021 to 31 August 2023, 69,679 newborns were screened for PID and SMA simultaneously. In this article, we excluded 23,216 tests for SMA, which were conducted as part of a pilot study of SMA-NBS conducted from 1 February 2021 to 30 September 2021 [27].

In the PID-NBS tests, 362 sample tests resulted in the testing of a new sample being ordered due to either inadequate PCR amplification caused by incomplete DNA extraction or the prematurity of the newborn. All of these patients were classified as normal after retests. Thirty-nine newborns exhibited abnormal TREC and/or KREC values. Twenty cases in which 0 copies of TREC were detected, three cases with low TREC copy numbers, eleven cases in which 0 copies of KREC were detected, one case with a low KREC copy number, and four cases in which 0 copies of TREC or KREC were detected were scrutinized.

One patient with a low KREC copy number was diagnosed with XLA. The patient, who was suspected to have BCD, underwent gene panel testing at the age of 34 days, leading to a diagnosis of XLA, which was confirmed by the detection of a known hemizygous missense mutation in the BTK gene. Immunoglobulin replacement therapy was initiated on day 75. The patient had not previously contracted any apparent infections, and all immunizations, including the rotavirus vaccine, were withheld.

In some of the cases with low KREC copy numbers, the patient’s mother had taken immunosuppressive agents, but in other cases no notable background factors were found. B lymphocytes were small at the first scrutiny examination, but their size subsequently normalized. Some of the newborns with low copy numbers of TREC or both TREC and KREC had health/developmental problems, including congenital heart disease, pleural effusion requiring drainage, Down syndrome, DiGeorge syndrome, or an extremely low birth weight. No cases of SCID have been found in our PID-NBS tests to date, although some cases are still being followed up carefully.

In the SMA-NBS tests, one screened case tested positive, and the other collected samples tested negative for *SMN1* deletion and did not require any further tests. There are three medical institutions in Osaka, including our center, that are capable of treating SMA patients with new drugs, including gene replacement therapy. Although we conducted a survey of the false-negative cases at these institutions, we found that there had not been any false-negative cases since the start of the SMA-NBS in Osaka. Thus, the sensitivity and specificity of our SMA-NBS tests were both estimated to be 100%.

The positive case was diagnosed with SMA through genetic testing based on the MLPA at 19 days after birth. Genetic testing showed the homozygous deletion of *SMN1* exon 7 and the presence of three *SMN2* copies. The patient was presymptomatic at the time of treatment and was treated with nusinersen and onasemnogene abeparvovec on day 21 and day 29, respectively. Although he showed mild hypotonia of his legs at the age of 7 months, he was able to walk independently, and his developmental milestones were within the normal range at 1 year and 3 months old.

## 4. Discussion

In our three-year experience of optional NBS based on a multiplex qPCR assay in Osaka, Japan, there have been no major problems with the NBS tests or programs, although our strategy involved starting with PID-NBS alone first and adding SMA-NBS fourteen months later. A case each of XLA and SMA have been diagnosed and treated at early stages (before symptoms appeared) through the optional NBS.

### 4.1. PID Screening in Osaka and the Merits of the Simultaneous Assessment of TREC and KREC Levels

We implemented NBS for PID in Osaka based on both KREC and TREC levels in 2020, making us one of the earliest groups in Japan to do so. However, the routine administration of the rotavirus vaccine in Japan was also started in 2020. In patients with SCID, the rotavirus vaccine, a live vaccine, has been reported to induce serious infections [28]. Therefore, screening for PID needs to be performed before patients reach vaccination age in order to prevent infectious diseases caused by live vaccines and to ensure that patients can acquire effective immunity with live or inactivated vaccines.

In NBS for PID in Osaka Prefecture, the proportion of retests that required a new sample was 0.3% (362/105,419). The previously reported retesting rates of 1.5% in Seville [29], 0.02% in Sweden [30], 0.13% at the Polish–German border [13], and 0.4% in Taiwan [31] are similar to our result. This suggests that the DNA extraction from the DBS and PCR testing were performed properly in our NBS program.

In addition, the percentage of positive cases that required further scrutiny in our NBS program was 0.037% (39/105,419). This was similar to the figures reported for California (the study with the highest number of cases) (0.017%; 562/3,252,156) [32], Seville (0.097%; 5/5160) [29], Sweden (0.11%; 64/58,834) [30], Iran (1.39%; 30/2160) [33], the Polish–German border (0.018%; 8/44,287) [13], and Taiwan (0.023%; 24/106,391) [31]. This suggests that our screening program exhibits similar levels of accuracy to those conducted in other countries.

Several pathologies other than severe PID were observed among the cases that underwent further examination after producing a positive result; i.e., an abnormal TREC and/or KREC value. The same diseases or conditions have been mentioned in previous studies [10,30,31,32]. Thus, many cases of inadequate immune function that do not correspond to XLA or SCID have been found by NBS. This indicates that such screening would make it possible to detect a variety of conditions that can cause lymphopenia. It is important to refrain from using live vaccines in patients with such conditions and to confirm the acquisition of immunity after vaccination. In extremely premature infants, the use of live vaccines is usually dependent on the patient’s chronological age rather than their corrected age, and it is better to vaccinate after lymphopenia has improved.

The sensitivity and specificity of our NBS program for detecting SCID/BCD were 100% and 99.96%, respectively. In Japan, a system has been established for registering new cases of PID. Therefore, it can be said that no false-negative cases have been observed so far. On the other hand, the positive predictive value of our NBS program for SCID/BCD was as low as 2.56%. However, this did not result in many false positives, and other causes of lymphopenia (or transient lymphopenia) were also captured. Thus, it can be said that that our program is useful as a screening test for lymphopenia.

The first advantage of simultaneously assessing TREC and KREC copy numbers is that it is possible to determine the underlying pathology in cases in which B lymphocytopenia arises alone. Furthermore, by combining TREC and KREC assessments it is possible to diagnose SCID, ataxia telangiectasia, and Nijmegen breakage syndrome and classify the disease type of common variable immunodeficiency [34]. In addition, it was previously reported that a case of IKZF1-related early-onset combined immunodeficiency was missed by TREC-based SCID screening but was identified when the patient’s KREC levels were determined [35]. Simultaneous TREC and KREC assessments are also useful for evaluating the reconstitution of the immune system after HSCT [36]. Thus, simultaneously assessing TREC and KREC levels has significant advantages.

### 4.2. SMA-NBS in Osaka and the Merits of Screening for PID and SMA Simultaneously

In 2019, initial discussions started about implementing an optional NBS in Osaka. In these discussions, PID was strongly recommended as a new target disease for NBS, and it was stated that PID-NBS had been successfully implemented on a large scale in several countries and some parts of Japan.

No problems were encountered with the implementation of PID-NBS in Osaka. However, ethical concerns about implementing SMA-NBS in Japan arose in 2019 due to it being the first genetic NBS program to directly detect genetic abnormalities and the lack of experience of gene replacement therapy for patients with SMA, which had just been approved in Japan. Therefore, implementing SMA-NBS and PID-NBS simultaneously in Osaka was difficult at that point. Ultimately, SMA-NBS was not considered to ethically deviate from the overall philosophy of NBS, and the efficacy and safety of the gene replacement therapy were deemed to be sufficient based on actual clinical practice, albeit according to short-term results (for a year after approval). The genetic ethical concerns raised regarding SMA-NBS were similar to a discussion in a previous study [37].

In Japan, SMA-NBS was first implemented in Chiba Prefecture, followed by Hyogo and Osaka. The multiplex qPCR assay used to detect PID and SMA was very useful for the additional implementation of SMA-NBS in Osaka. In particular, the cost-effectiveness and labor-saving effects of this approach were considered to be great advantages. The actual cost of adding SMA-NBS to an NBS assay for PID was estimated to be USD 0.05 per test in our NBS program. Therefore, we were able to implement SMA-NBS without having to charge any additional fees. Tesorero R et al. also reported on the scalability and cost-effectiveness of multiplex qPCR assays by integrating screening for sickle cell disease into a pre-existing duplex NBS assay for SCID and SMA [38]. In addition, as the basis for optional NBS in Osaka had been established by the preceding PID-NBS, the obstetricians in the region had gained a deeper understanding of other treatable target diseases for NBS, including SMA, that should be diagnosed in the neonatal period. This was considered to be one of the reasons why SMA-NBS was added smoothly in Osaka.

A previous study reported that there are several countries that are still having some difficulties with the implementation of SMA-NBS, such as a lack of financial resources and governmental support or a lack of long-term follow-up data on specific treatments for SMA or cost–benefit analysis data [39]. Approving very expensive drugs for SMA may also contribute to these difficulties. However, SMA-NBS has already been recognized as practical and effective in many countries, and more countries and local governments are expected to plan to implement SMA-NBS in the near future [40,41,42,43]. Therefore, first implementing PID-NBS based on a multiplex qPCR assay and then adding SMA-NBS after resolving its implementation difficulties in each area, as we did in Osaka, is considered to be a useful strategy for the further dissemination of SMA-NBS.

### 4.3. Issues with PID-NBS and SMA-NBS

Performing NBS using a multiplex qPCR assay is a cost-effective and well-established system. Furthermore, the number of target diseases for NBS that can be detected by PCR-based screening, such as congenital cytomegalovirus infections, is expected to increase in the near future [44,45]. NBS programs based on multiplex qPCR assays have further scalability. However, there are still some issues that need to be resolved for each new target disease, including PID and SMA.

#### 4.3.1. PID-NBS

In the future, it will be necessary to examine the prognosis and medical costs of patients with PID that are identified by NBS and compare them with those of patients with PID that are identified based on their family history and infectious diseases. In addition, it will be necessary to build a patient registration system for PID in Japan in order to reliably identify patients who develop PID and evaluate the sensitivity and specificity of NBS for PID.

#### 4.3.2. SMA-NBS

Regarding the issues with SMA-NBS, no method for finding SMA patients with compound heterozygous abnormalities involving unilateral allelic deletion and unilateral allelic point mutation of SMN1 (which is seen in approximately 5% of SMA patients), which are not targeted by current SMA-NBS testing, has yet been established. Although SMN1 copy number analysis using qPCR and DBS samples is technically possible, it would be complicated as a screening test and would raise ethical concerns, as it would involve carrier screening [46]. Therefore, it is also important to make public and healthcare professionals aware that SMA cannot be completely ruled out even when SMA-NBS produces a negative result. Second, the cost-effectiveness of SMA-NBS has been debated, as the current drugs for patients with SMA are very expensive. Most previous studies reported that if early (presymptomatic) treatment can be achieved via SMA-NBS, then the cost-effectiveness of SMA-NBS would be improved [47,48,49,50]. Although there are no studies on the cost-effectiveness of SMA-NBS in Japan, the Japanese Society of Child Neurology is currently working on estimates of its cost-effectiveness in Japan. Third, since there is still a lack of long-term follow-up data on patients with SMA who were diagnosed and treated early through NBS, it is necessary to accumulate and evaluate actual clinical data, such as about the efficacy and safety of treatment and whether NBS contributes to improving the quality of life of patients with SMA. As there is still no large-scale registration system for SMA-NBS in Japan, the establishment of a long-term follow-up system for patients with SMA is an urgent issue.

### 4.4. Limitations of Our Project and Optional NBS Programs in Japan

After three years of data accumulation, the incidence rate of XLA in our program is approximately 1 in 100,000 births, and no cases of SCID have been identified. The incidence of XLA in our program seems reasonable; however, the incidence rates of SCID in our optional NBS program are lower than previously reported rates [51]. There may be regional and/or racial differences in the incidence rates of these diseases. On the other hand, the incidence of SMA in Osaka (approximately 1/69,000 births) is extremely low. The reported incidences of SMA, which were derived from several years of statewide or nationwide pilot studies or universal screening, range from 1/6000 to 1/19,000 births [25,40,41,42,43,52]. The reasons for this wide range of incidence rates are considered to include differences in study size and duration and the sociocultural background regarding prenatal genetic testing, such as whether carrier screening programs are routinely performed. In a nationwide epidemiological survey conducted in Japan, Ito M et al. reported that the incidence of SMA was approximately 1/20,000 births, although they observed regional differences in the incidence of SMA [53]. There have only been two studies of SMA-NBS in Japan in places other than Osaka, and they reported short-term SMA-NBS incidence rates of 3/16,037 births for a 2-year study conducted in Hyogo and 1/13,587 births for a 1-year study conducted in Kumamoto [54,55], although these studies also differed in size. The cause of our low SMA incidence is unclear at this time. A longer study involving a higher participation rate for SMA-NBS is needed to accurately evaluate the incidence of SMA in Osaka.

In Japan, optional NBS programs are mainly managed by the NBS center in each area and are based on parents’ voluntary participation. They require payment, as they are not funded by the government, since there is no new target disease early inclusion system for universal NBS in Japan similar to RUSP in the US. However, fee-based optional NBS programs are unfair to parents. By August 2023, the optional NBS programs run by OWCH covered about 80% of the newborns in Osaka. Although more effort is needed to increase the participation rate of the optional NBS programs in Osaka, fees can introduce hard limits to participation. Therefore, it will be necessary to quickly establish criteria for adding new target diseases to the universal NBS program in Japan. Moreover, improving the prognosis of patients with rare genetic diseases through innovative treatments, such as gene therapy, has led to a need for a long-term follow-up system and genetic counseling provided by people with appropriate knowledge about each disease [56]. Securing enough genetic counselors in each area may become a problem in the near future. It will also be necessary to train genetic counselors who have appropriate knowledge about current NBS systems.

## 5. Conclusions

Drawing from the positive three-year outcomes of the optional NBS program in Osaka, Japan, as detailed in this article, we recommend the broader implementation of NBS for PID and SMA in other countries and regions. The success of our program, which was characterized by effective treatment and improved prognoses being achieved through early detection, suggests a promising model for others to consider. Moreover, NBS using DNA extracted from DBS and a multiplex qPCR assay is considered to be highly accurate, cost-effective, and scalable and could be used for new target diseases in the future. Although adding new target diseases to an existing system requires additional effort and cost, selecting diseases that can be screened for simultaneously using multiplex qPCR can be effective in reducing these problems. Therefore, the stepwise addition of new target diseases is also considered to be a useful strategy for NBS using multiplex qPCR assays.

## Data Availability

The data presented in this study are available on reasonable request from the corresponding author. The data are not publicly available in order to maintain confidentiality.
